# Rhodococcus induced false-positive galactomannan (GM), a biomarker of fungal presentation, in patients with peritoneal dialysis: case reports

**DOI:** 10.1186/s12882-019-1642-1

**Published:** 2019-12-02

**Authors:** Tamonwan Chamroensakchai, Wasin Manuprasert, Asada Leelahavanichkul, Kullaya Takkavatakarn, Nisa Thongbor, Bunpring Jaroenpattrawut, Talerngsak Kanjanabuch

**Affiliations:** 1 Department of Microbiology, Faculty of Medicine, Chulalongkorn University, Bangkok, Thailand; 2 Center of Excellence in Kidney Metabolic Disorders, Faculty of Medicine, Chulalongkorn University, Bangkok, Thailand; 3 Division of Nephrology, Department of Medicine, Faculty of Medicine, Chulalongkorn University, Bangkok, Thailand; 4 Department of Medicine, Sunpasitthiprasong Hosptial, Ubon Ratchathani, Thailand; 5 Continuous Ambulatory Peritoneal Dialysis Clinic (CAPD), Nakhon Pathom Hospital, Nakhon Pathom, Thailand; 6 CAPD Excellent Center, King Chulalongkorn Memorial Hospital, Bangkok, Thailand

## Abstract

**Background:**

Galactomannan index (GMI) at a level higher than 0.5 provides high sensitivity and specificity for the diagnosis of fungal peritonitis. Here, we report the false-positive of GMI in peritoneal dialysis (PD) effluent (PDE) due to *Rhodococcus* peritonitis in PD patients.

**Case presentation:**

GMI in PDE of case #1 and case #2 were 1.53 and 0.76, respectively, while serum GMI of both cases was less than 0.5. In addition, GMI from the specimens obtained directly from the stationary phase of *Rhodococcus* colonies were 1.27 and 1.56, which were isolated from case #1 and #2, accordingly.

**Conclusion:**

High GMI in PDE of PD patients is not specific just for fungal infections but may also be secondary to other infections, such as *Rhodococcus* spp., especially in endemic areas.

## Background

Fungal peritonitis is a relatively uncommon but serious complication of peritoneal dialysis (PD) and is associated with a high risk of technique failure and mortality, especially in cases of delayed diagnosis. The galactomannan (GM) test is an easier technique for the detection of fungal cell wall components shedding out of the fungi during fungal growth and death [1]. Indeed, GM index (GMI), analyzed by a sandwich-ELISA (Bio-Rad Platelia™, USA), has been widely used for the diagnosis of systemic fungal infection [2] and has been recently proposed as an alternative marker of fungal peritonitis [3]. Despite the high sensitivity and specificity of GMI in PD effluent (PDE) for the diagnosis of fungal peritonitis [3] and fungal colonization inside a PD catheter [4], we reported a false-positive GMI in PDE from patients infected with *Rhodococcus* spp. There were 2 patients with rhodococcal infection who had orange stains inside their PD catheters. Both cases were mistakenly diagnosed as fungal peritonitis and resulting in initial treatment failure.

## Case presentation

Case #1 was a 71 year-old man with diabetic end-stage renal disease (ESRD) who was performing continuous ambulatory PD (CAPD) at Sunpasitthiprasong Hospital since 2013. On 30 July 2017 (day 0), he presented with acute abdominal pain and cloudy dialysate associated with a normal exit-site. The diagnosis of peritonitis was confirmed with a PDE leukocyte count of 2900 cells/μL of which 91% were neutrophils. The empirical therapy for bacterial peritonitis with intraperitoneal (IP) cefazolin 1 g together with ceftazidime 1 g IP once daily was commenced. Although the dialysate leukocyte gradually decreased, the leukocyte persisted at more than 100/μL with neutrophil predominance. The dialysate from day 0 failed to culture any organism. However, many orange spots were noticed inside the PD catheter on day + 8. GMI in the PDE and the sera were 1.53 (normal< 0.5) and 0.39 (normal< 0.5), respectively. The provisional diagnosis of fungal peritonitis was made. Intravenous amphotericin B, 0.5 mg/kg/day was promptly started on the following day (day + 9), and the PD catheter was subsequently removed on day + 11. The PD catheter, serum, and drained PDE were submitted to the central microbiology lab for microorganism identification. The patient was transferred to hemodialysis (HD) following PD catheter removal. The patient continued to improve while having intravenous (IV) amphotericin and ceftazidime that were prescribed for a total of 2 weeks.

Case #2, a 59 year-old man with a past medical history of diabetes and hypertension, was diagnosed with ESRD from diabetes. He commenced CAPD (4 exchanges/day) in January 2017 at Nakhon Pathom Hospital. While performing CAPD, he was able to maintain adequate dialysis small solute clearance and had never experienced peritonitis. On 16 January 2018 (day 0) the patient presented with cloudy dialysate, abdominal pain, poor appetite, and ultrafiltration failure, associated with a normal exit-site. The PDE cell count was 497 cells/μL with a neutrophil predominance (63%). He was initially diagnosed with CAPD-related peritonitis and received a combination of IP cefazolin and ceftazidime on the same day resulting in partial resolution of the abdominal pain. However, the PDE leukocyte count that was repeated on day + 3 indicated an increased count of 2080 cells/μL (85% neutrophils), while the dialysate culture from day 0 later yielded *Corynebacterium* spp. The primary physician swapped the antibiotics to IV vancomycin, 1 g every 3 days, and IP amikacin, 25 mg loading dose followed by 12 mg once daily for a total duration of 2 weeks. During the follow-up period, salmon-pink colonies were observed inside the PD catheter and GMI in PDE from day + 10 was later reported positive at a level of 0.76 (< 0.5). Superimposed fungal peritonitis was suspected, although IP amphotericin B was added on day + 13, and the PD catheter was removed on day + 16. Amphotericin was later withdrawn after a return of culture report from the central microbiology lab on day + 19 demonstrated *Rhodococcus equi*. Temporary HD was performed during day + 17 to day + 56. The PD catheter was reinserted on day + 58, and the patient has remained well on CAPD without any subsequent peritonitis.

**Fig. 1:**
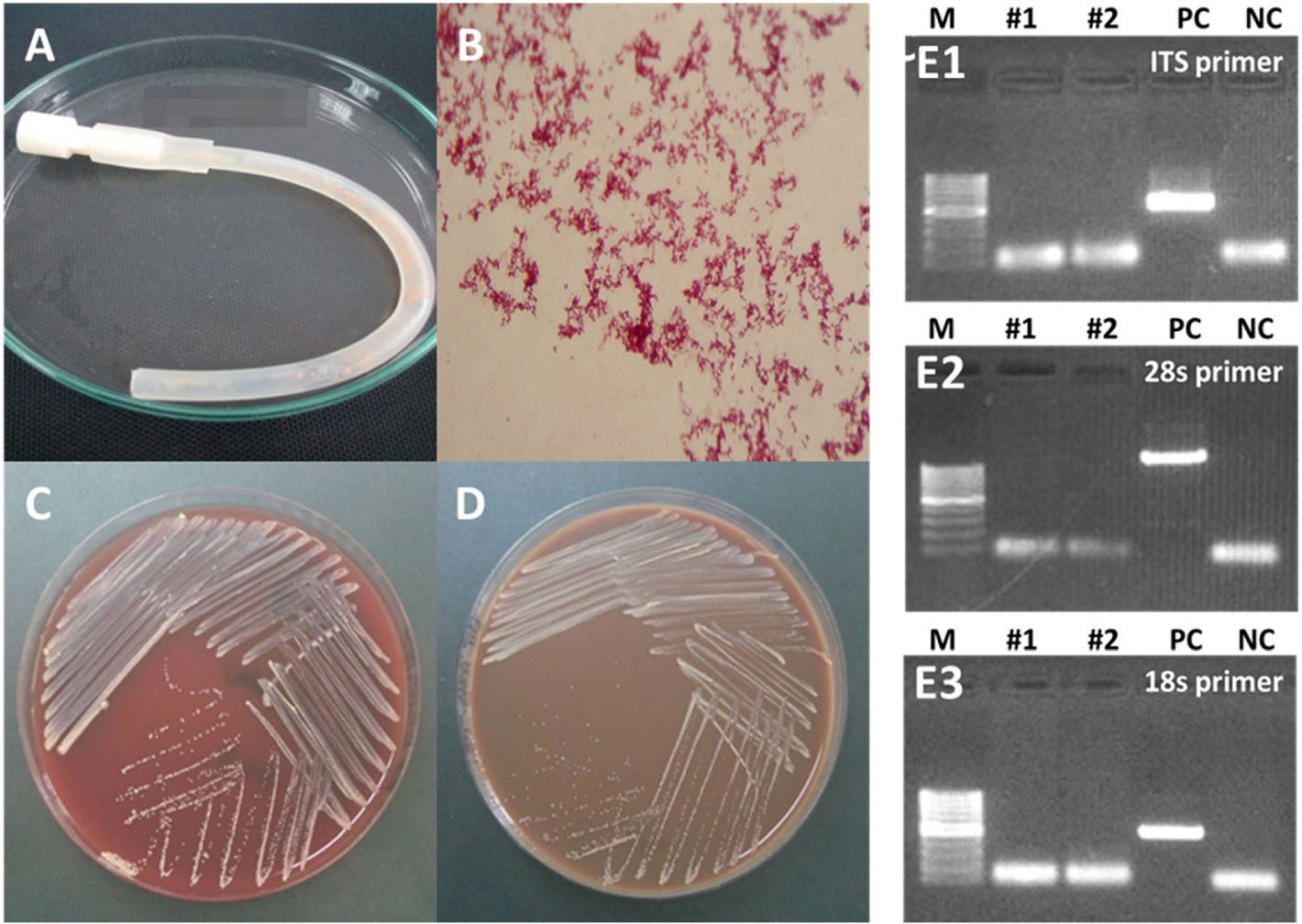
The orange stains on (**a**) PD catheter. **b** Modified acid-fast stain of TS catheter specimen depicts numerous microorganisms. The orange colony was grown on (**c**) Blood agar, and (**d**) chocolate agar after 72 h. Fungal co-infection was excluded by various fungal primers, including ITS (E1), 28S (E2), and 18S (E3) primers. Abbreviations: NC, negative control; PC, positive control

At the central microbiology lab, the removed catheters and the PDE were submitted for culture in several media. Interestingly, the orange colonies on tryptic soy agar (TSA) and chocolate agar were observed at 72 h, but no colony growth on SDA (Fig. 1). *Rhodococcus* spp. and *Rhodococcus equi* was identified by modified AFB and the biochemistry assay from case #1 and #2, respectively. The GMI in the specimens obtained directly from colonies of *Rhodococcus* were 1.27 (Case #1) and 1.58 (case #2), which were above the cut-off value (≥ 0.5) using a GM test kit (Bio-Rad Platelia™, USA).

## Discussion and conclusion

*Rhodococcus*, a gram-positive filamentous bacteria which belongs to phylum Actinobacteria, causes disease only in immune-compromised hosts [5]. This organism can survive in various habitats such as terrestrial (soil) and aquatic environments (fresh and saltwater) [6, 7]. Fungal co-infection was excluded from the presented cases using both fungal culture and PCR with universal fungal primers (16S, 28S, and ITS) (Fig. 1e). Although *Rhodococcus* is sensitive to several antibiotics [6], early catheter removal was performed in both cases because of the misdiagnosis of fungal peritonitis by the primary physicians. The ISPD Guideline 2016 recommends an immediate PD catheter removal when fungi are identified [8]. Thailand has a high incidence of culture-negative peritonitis, particularly in provincial hospitals. The false-positive GMI caused by this infection has never been mentioned despite the reported lower sensitivity and specificity for the GM test (77 and 58% respectively) [3].

The cell envelop of the *Rhodococcus* does not express GM, it contains a substantial amount of arabinogalactan [AG: a linear galactan of galactofuranose (Galf) residues + discrete arabinan side branches which contain arabinofuranoses) and lipoarabinomannan (LAM: lipoglycan core + mannan side chains) of which both are bound to branched long-chain lipids termed mycolic acids. The galactan of *R. equi* contains a mixture of (1 → 3), (1 → 5), and (1 → 6) linkages [7]. On the other hand, GM found in pathologic filamentous fungi is a hetero-polysaccharide consisting of a linear-mannan core with a repeating tetramannose unit (2 M–6 M-2 M-2 M) and its side chains (4–5 residues of β-(1 → 5)-Galf attached to mannose) [9, 10]. Both organisms contain β-(1 → 5)-Galf which is a target for the immune system and monoclonal antibody using in the commercial GM test kits (Bio-Rad Platelia™, USA and Pastorex Aspergillus™, Diagnostic Pasteur, France) [9, 10]. However, the Galf is located in a different strand of carbohydrate heteropolymer where filamentous mold is on the side chain of GM while the *Rhodococcus* is on the backbone of AG [9].

Since there are very few case reports of rhodococcal peritonitis, experiences of the preferred treatment are limited. A combination of antibiotics is the mainstay of treatment, especially antibiotics with intracellular activity against the organism. Although *Rhodococcus* is initially sensitive to cephalosporins, it is not recommended in clinical practice because of the potential development of late resistance [11]. The duration of treatment in immunocompetent patients may range from 2 to 8 weeks or even longer depending upon the severity of the disease [12]. Removal of the PD catheter should be considered in *Rhodococcus* peritonitis due to the unpredictable response of this organism to treatment [13]. Our 2 cases demonstrated the success of management of *Rhodococcus* peritonitis with 2 weeks of antibiotics combined with the PD catheter removal. Case #2 was treated with IP vancomycin plus amikacin. Meanwhile, case #1 was successfully treated by IP ceftazidime. The *Rhodococcus* infections in both of these cases either responded to the empirical treatment or PD catheter removal.

In conclusion, false-positive GMI results may occur with *Rhodococcus* infection. Thus, various culture media and polymerase chain reaction should be used to confirm the results of high GMI in PDE, especially in endemic areas of rhodococcal infection.

## Data Availability

The data that support the findings of this study are available from the corresponding author upon reasonable request.

## Abbreviations

AFB: Acid-fast stain
AG: Arabinogalactan
CAPD: Continuous ambulatory peritoneal dialysis
ESRD: End-stage renal disease
GM: Galactomannan
GMI: Galactomannan index
HD: Hemodialysis
IP: Intraperitoneal
ISPD: International Society for Peritoneal Dialysis
IV: Intravenous
LAM: Lipoarabinomannan
PCR: Polymerase chain reaction
PD: Peritoneal dialysis
PDE: Peritoneal dialysis effluent
SDA: Sabouraud dextrose agar
TSA: Tryptic soy agar
